# Comparison of the effect of continuous intravenous infusion and two bolus injections of remifentanil on haemodynamic responses during anaesthesia induction: a prospective randomised single-centre study

**DOI:** 10.1186/s12871-016-0275-1

**Published:** 2016-11-14

**Authors:** Toyoaki Maruta, Yoshihumi Kodama, Ishie Tanaka, Tetsuro Shirasaka, Isao Tsuneyoshi

**Affiliations:** 1 Department of Anesthesiology, Miyazaki Medical College, University of Miyazaki, Miyazaki, 889-1692 Japan; 2 Department of Anesthesiology, Miyazaki Medical Association Hospital, Miyazaki, 880-0834 Japan; 3 Department of Internal Medicine, Koga General Hospital, Miyazaki, 880-0041 Japan

**Keywords:** Remifentanil, Anaesthesia induction, Laryngoscopy, Intubation, Prospective study

## Abstract

**Background:**

Remifentanil is an effective drug for protecting against adverse haemodynamic responses to tracheal intubation. We compared the haemodynamic responses during anaesthesia induction between continuous intravenous (IV) infusion and two bolus injections of remifentanil.

**Methods:**

This prospective, randomised, open-label, single-centre study included patients with American Society of Anesthesiologists physical status I-II, scheduled to undergo elective surgery under general anaesthesia. Patients were randomised into two groups based on remifentanil administration type: the continuous IV infusion group (Group C) receiving a 0.3-μg/kg/min remifentanil infusion for 5 min followed by a 0.1-μg/kg/min remifentanil infusion, and the IV bolus group (Group B) receiving a combination of two bolus injections of remifentanil (first bolus of 0.4 μg/kg and second bolus of 0.6 μg/kg after 3 min) and 0.1 μg/kg/min remifentanil. General anaesthesia was induced with 1 mg/kg propofol and 0.6 mg/kg rocuronium 3 min after remifentanil infusion (Group C) or immediately after the first bolus of remifentanil (Group B). Tracheal intubation was performed 4 min after the injection of propofol and rocuronium. Heart rate and non-invasive blood pressure were recorded at 1-min intervals from baseline (i.e., before induction) to 5 min after tracheal intubation.

**Results:**

A total of 107 patients were enrolled (Group C, 55; Group B, 52). Normotensive patients with no history of antihypertensive medication use were assigned to the normotensive subgroup (41 each in both groups), while those with hypertension but without a history of antihypertensive medication use were assigned to the untreated hypertensive subgroup (Group C vs. B, *n* = 7 vs. 4). Finally, patients with a history of antihypertensive medication use were assigned to the treated hypertensive subgroup (7 each in both Groups C and B). No differences in heart rate and blood pressure were observed between Groups C and B within each subgroup.

**Conclusions:**

Haemodynamic responses during anaesthesia induction were similar between continuous infusion and two bolus injections of remifentanil within both normotensive and hypertensive patients with or without medication.

**Trial registration:**

The trial was retrospectively registered with Japanese Clinical Trial Registry “UMIN-CTR” on 20 October 2016 and was given a trial ID number UMIN000024495.

## Background

Remifentanil is a rapid-onset and ultra-short acting opioid [1–3]. Since laryngoscopy and tracheal intubation can result in tachycardia and hypertension, remifentanil is commonly used not only to maintain anaesthesia, but also to attenuate adverse haemodynamic changes due to tracheal intubation [4–6]. To raise the effect-site concentration of remifentanil quickly, it is infused at higher concentrations or injected in bolus doses prior to constant continuous infusion. However, differences in haemodynamics during anaesthesia induction between continuous infusion and bolus injection of remifentanil have not yet been investigated. In addition, it remains unclear which method is best suited to hypertensive patients in whom tachycardia and hypertension by intubation may be exaggerated.

The aims of this randomised open-label study were to compare the haemodynamic responses during anaesthesia induction between continuous intravenous infusion and bolus injections of remifentanil in normotensive patients, as well as in treated and untreated hypertensive patients.

## Methods

### Ethics and study design

This study was approved by the hospital ethics committee for human studies (Ethical Committee number 2013–079; Chairperson Professor Koichiro Itai) on 3 October 2013, and all patients provided informed written consent. American Society of Anesthesiologists physical status (ASA) I-II patients scheduled for elective surgery under general anaesthesia were prospectively enrolled. Exclusion criteria included patients younger than 20 years of age and with the presence of cardiovascular or cerebrovascular disease, renal failure, or a predicted difficult airway. If tracheal intubation was not performed within 1 min, this patient was excluded.

**Fig. 1:**
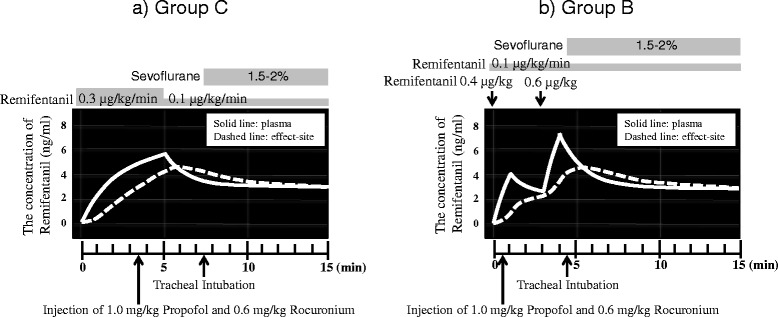
The time course of plasma and effect-site concentration of remifentanil in Group C (**a**) and Group B (**b**). The plasma and effect-site concentrations of remifentanil were calculated using the Egan model, which was a default formula used in PrimeGaia, the electronic anaesthesia record

Normotensive patients with no history of antihypertensive medication use were assigned to the normotensive patients subgroup, while those with hypertension (systolic blood pressure (SBP) > 140 mmHg) were assigned to the untreated hypertensive (HT) patient subgroup. Hypertensive patients with a history of antihypertensive medication use were assigned to the treated HT patients subgroup. Using a stratified permuted block randomization method, normotensive patients were randomized to two groups of remifentanil administration type; the continuous intravenous (IV) infusion group (Group C) and the IV bolus group (Group B). Group C patients received a 0.3 μg/kg/min infusion of remifentanil for five minutes followed by a 0.1 μg/kg/min infusion of remifentanil, and Group B patients received a combination of two bolus injections of remifentanil (first bolus of 0.4 μg/kg and second bolus of 0.6 μg/kg in a 3 min interval) with 0.1 μg/kg/min remifentanil. The treated HT and untreated HT patients were also randomized into two groups using a simple randomization method by random number tables. The time courses of anaesthesia induction in Group C and B are shown in Fig. 1. General anaesthesia was induced using 1 mg/kg propofol and 0.6 mg/kg rocuronium 3 min after infusion of remifentanil in Group C or immediately after the first bolus of remifentanil in Group B. An additional 10 mg of propofol was administrated every 10 s until there was a loss of verbal response. Tracheal intubation was performed 4 min after induction of anaesthesia (7 min after the infusion of remifentanil in Group C or 4 min after the first bolus of remifentanil in Group B), at which estimated effect-site concentration of remifentanil was approximately 4 ng/ml in each group. After intubation, general anaesthesia was maintained with 1.5–2 % inspired sevoflurane and 0.1 μg/kg/min remifentanil in both groups.

### Measurements

Heart rate (HR) and non-invasive blood pressure (NIBP) were recorded at 1-min intervals from baseline (prior to induction) to 5 min after tracheal intubation. NIBP every 1 min is not always possible due to time taken for each measurement and has the potential to affect subsequent mesurements. However, it was considered that invasive blood pressure measurement was not suitable for this study, because almost patients were performed minor surgeries. If NIBP measurement was failed, this patient was excluded. If HR or SBP decreased to less than 50 bpm or 70 mmHg respectively, IV of 4 mg ephedrine was deemed necessary. The estimated effect-site concentration of remifentanil was calculated with the Egan model [7] in the electronic Anaesthesia record “PrimeGaia” (Nihon Kohden, Tokyo, Japan).

### Statistical analysis

A prior sample size calculation was performed, which revealed that 30 patients per group would have an 80 % power with a *p* < 0.05 to detect 15 mmHg differences in mean arterial pressure between groups (α = 0.05, β = 0.2). All data are presented as mean ± standard deviation (SD), numbers or percentage. Statistical analysis was performed using the program JMP 11 (SAS Institute Inc, Cary, NC, USA). Categorical data were examined using a chi-square test or Fisher’s exact test to compare groups. One-way ANOVA for repeated measures was used to analyse changes over time. Significance (*p* < 0.05) was determined by one-way ANOVA with post hoc mean comparison by the Tukey-Kramer honestly significant difference (HSD) test. The student’s *t* test was used when means of two groups were compared.

## Results

A total of 107 patients were recruited from October 2013 to June 2015. Patient characteristics are shown in Table 1. There were 82 normotensive patients older than 20 years of age (*n* = 41 in both groups), 14 treated HT patients (*n* = 7 in both groups) and 11 untreated HT patients (*n* = 7 in Group C and *n* = 4 in Group B). Both the treated and untreated HT subgroups were compared between Group C and Group B. In both normotensive and treated HT patients, there were no significant differences between Group C and Group B in age, gender, height, weight, BMI, and ASA (Table 1). Anti-hypertensive medications which treated hypertension patients were taking are shown in Table 2. Ca blocker was continued through the morning of surgery, and angiotensin II receptor blocker was continued through the day before surgery. There were no significant differences between Group C and Group B in anti-hypertensive medications.

**Table 1:** Data characteristics of patients, stratified by group

	Group C			Group B		
Subgroups	Normotensive (*n* = 41)	Treated HT (*n* = 7)	Untreated HT (*n* = 7)	Normotensive (*n* = 41)	Treated HT (*n* = 7)	Untreated HT (*n* = 4)
Age (year)	46 ± 16	66 ± 9	57 ± 13	45 ± 16	67 ± 5	63 ± 7
Male/Female	21/20	4/3	2/5	21/20	7/0	2/2
Height (cm)	161 ± 8	158 ± 8	155 ± 6	162 ± 8	162 ± 13	160 ± 15
Weight (kg)	57 ± 10	59 ± 9	55 ± 5	60 ± 9	66 ± 13	57 ± 11
BMI	21.8 ± 3.1	23.7 ± 3.5	22.7 ± 2.8	22.7 ± 2.8	24.8 ± 2.9	22.3 ± 1.0
ASA I/II	28/13	0/7	0/7	27/14	0/7	0/4

The results are expressed as mean ± SD or numbers of patients. Normotensive: patients with no history of antihypertensive medication use and with normotension, treated HT: patients with history of antihypertensive medication use, untreated HT: patients with no history of antihypertensive medication use and with hypertension, *HT* hypertensive, *BMI* body mass index, *ASA* American Society of Anesthesiologists physical status

**Table 2:** Antihypertensive medication in treated hypertensive patients

	Group C	Group B
Subgroups	Treated HT (*n* = 7)	Treated HT (*n* = 7)
Ca blocker	5 (72 %)	3 (42 %)
ARB	1 (14 %)	2 (29 %)
Ca blocker + ARB	1 (14 %)	2 (29 %)

The results are expressed as numbers and %. Ca blocker was continued through the morning of surgery, and ARB was continued through the day before surgery. Treated HT: patients with a history of antihypertensive medication use, *HT* hypertensive, *ARB* angiotensin II receptor blocker

### Comparison of haemodynamic changes during anaesthesia induction between Group C and Group B

**Fig. 2:**
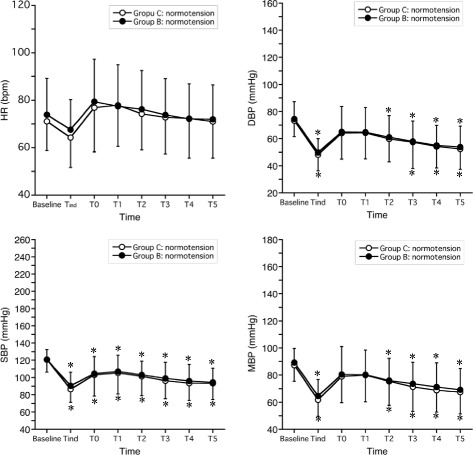
Heart rate (HR), systolic blood pressure (SBP), diastolic blood pressure (DBP), and mean blood pressure (MBP), stratified by group, for normotensive patients (mean ± SD). Haemodynamic variables were recorded before induction of general anaesthesia (baseline), immediately before tracheal intubation (T_ind_), immediately after tracheal intubation (T0), and every minute for 5 min following tracheal intubation (T1-T2). *p < 0.05, compared with baseline within each group

Comparisons of HR, SBP, diastolic blood pressure (DBP), and mean blood pressure (MBP) in normotensive patients between Group C and Group B are shown in Table 3 and illustrated in Fig. 2. There were no significant differences between the groups for any parameters at any time points. When compared to baseline, HR was not significantly different at any time points in either group; however, upon comparison between immediately before tracheal intubation (T_ind_) and immediately after tracheal intubation (T0), SBP, DBP, and MBP were significantly decreased compared to baseline in each group.

**Table 3:** Haemodynamic data during anaesthesia induction

Subgroups		Normotensive		Treated HT		Untreated HT	
		Group C (*n* = 41)	Group B (*n* = 41)	Group C (*n* = 7)	Group B (*n* = 7)	Group C (*n* = 7)	Group B (*n* = 4)
HR (bpm)	Baseline	70 ± 8	74 ± 13	68 ± 19	77 ± 12	74 ± 23	70 ± 10
	T_ind_	62 ± 10	67 ± 10	59 ± 9	64 ± 11	61 ± 17	62 ± 10
	T0	77 ± 20	80 ± 16	72 ± 15	68 ± 13	66 ± 11	87 ± 18
	T1	76 ± 13	77 ± 12	72 ± 15	82 ± 16	74 ± 20	80 ± 15
	T2	72 ± 10	74 ± 13	68 ± 14	78 ± 18	69 ± 19	73 ± 14
	T3	70 ± 9	71 ± 11	69 ± 13	72 ± 17	66 ± 16	70 ± 12
	T4	68 ± 10	70 ± 11	67 ± 11	70 ± 13	66 ± 17	66 ± 9
	T5	67 ± 10	70 ± 10	66 ± 11	71 ± 19	61 ± 13	68 ± 9
SBP (mmHg)	Baseline	123 ± 14	122 ± 13	145 ± 19	136 ± 19	153 ± 9	154 ± 11
	T_ind_	81 ± 18*	80 ± 12*	79 ± 16*	80 ± 21*	93 ± 14*	100 ± 16*
	T0	92 ± 30*	100 ± 17*	97 ± 20*	98 ± 26*	109 ± 36*	153 ± 40
	T1	96 ± 25*	107 ± 18*	107 ± 23*	124 ± 41	133 ± 42	147 ± 15
	T2	94 ± 25*	101 ± 15*	103 ± 22*	122 ± 46	123 ± 42	151 ± 46
	T3	88 ± 18*	96 ± 20*	104 ± 21*	115 ± 30	112 ± 40*	123 ± 17
	T4	86 ± 16*	93 ± 20*	96 ± 15*	109 ± 26*	111 ± 37*	116 ± 21*
	T5	87 ± 17*	90 ± 17*	95 ± 15*	102 ± 19*	106 ± 31*	115 ± 10*
DBP (mmHg)	Baseline	76 ± 14	77 ± 14	82 ± 19	82 ± 7	86 ± 9	92 ± 11
	T_ind_	47 ± 14*	49 ± 10*	48 ± 15*	51 ± 9*	50 ± 7*	66 ± 17*
	T0	61 ± 23	66 ± 17	62 ± 17	65 ± 13*	70 ± 32	99 ± 25
	T1	62 ± 23	69 ± 16	68 ± 16	75 ± 22	75 ± 31	96 ± 21
	T2	58 ± 19	66 ± 17*	62 ± 14*	74 ± 20	70 ± 27	86 ± 26
	T3	55 ± 16*	62 ± 16*	61 ± 12*	68 ± 15*	63 ± 24*	77 ± 17
	T4	53 ± 14*	59 ± 17*	58 ± 10*	68 ± 15*	60 ± 21*	73 ± 16
	T5	52 ± 12*	57 ± 15*	57 ± 10*	62 ± 7*	57 ± 19*	72 ± 11*
MBP (mmHg)	Baseline	90 ± 11	89 ± 10	101 ± 29	97 ± 6	105 ± 12	114 ± 14
	T_ind_	58 ± 12*	59 ± 10*	57 ± 15*	59 ± 14*	66 ± 11*	73 ± 19*
	T0	73 ± 22	78 ± 19	73 ± 17*	73 ± 16*	82 ± 31	115 ± 32
	T1	72 ± 18	79 ± 16	80 ± 17	92 ± 27	92 ± 36	111 ± 14
	T2	68 ± 16*	77 ± 18*	75 ± 17	91 ± 36	88 ± 30	100 ± 31
	T3	65 ± 14*	73 ± 16*	72 ± 15*	83 ± 21	81 ± 24*	89 ± 20
	T4	64 ± 12*	71 ± 20*	69 ± 11*	78 ± 15*	80 ± 26*	88 ± 15*
	T5	62 ± 11*	69 ± 16*	68 ± 10*	74 ± 12*	76 ± 20*	87 ± 13*

The results are expressed as mean ± SD. Haemodynamic variables were recorded before induction of general anaesthesia (baseline), immediately before tracheal intubation (T_ind_), immediately after tracheal intubation (T0), and every minute for 5 min following tracheal intubation (T1-T5)

*p < 0.05, compared with baseline within each group

*HR* heart rate, *SBP* systolic blood pressure, *DBP* diastolic blood pressure, *MBP* mean blood pressure

**Fig. 3:**
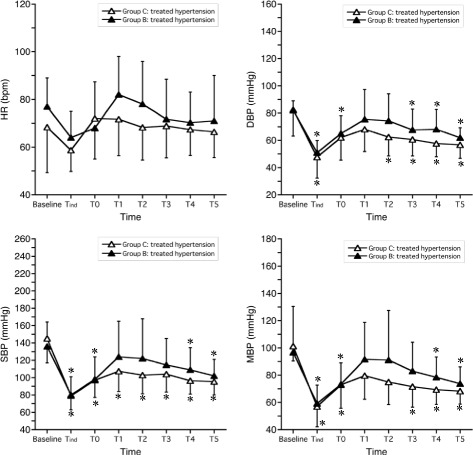
Heart rate (HR), systolic blood pressure (SBP), diastolic blood pressure (DBP), and mean blood pressure (MBP), stratified by group, for treated hypertensive patients (mean ± SD). Haemodynamic variables were recorded before induction of general anaesthesia (baseline), immediately before tracheal intubation (T_ind_), immediately after tracheal intubation (T0), and every minute for 5 min following tracheal intubation (T1-T2). *p < 0.05, compared with baseline within each group

**Fig. 4:**
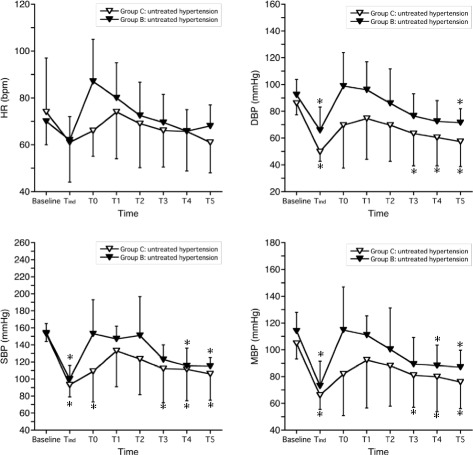
Heart rate (HR), systolic blood pressure (SBP), diastolic blood pressure (DBP), and mean blood pressure (MBP), stratified by group, for untreated hypertensive patients (mean ± SD). Haemodynamic variables were recorded before induction of general anaesthesia (baseline), immediately before tracheal intubation (T_ind_), immediately after tracheal intubation (T0), and every minute for 5 min following tracheal intubation (T1-T5). *p < 0.05, compared with baseline within each group

Comparisons of HR, SBP, DBP, and MBP within treated and untreated HT patients between Group C and Group B are shown in Table 3 and illustrated in Figs. 3 and 4, respectively. There were no significant differences between groups for any parameters at any time points in each subgroup.

### Use of additional propofol and adverse events

**Fig. 5:**
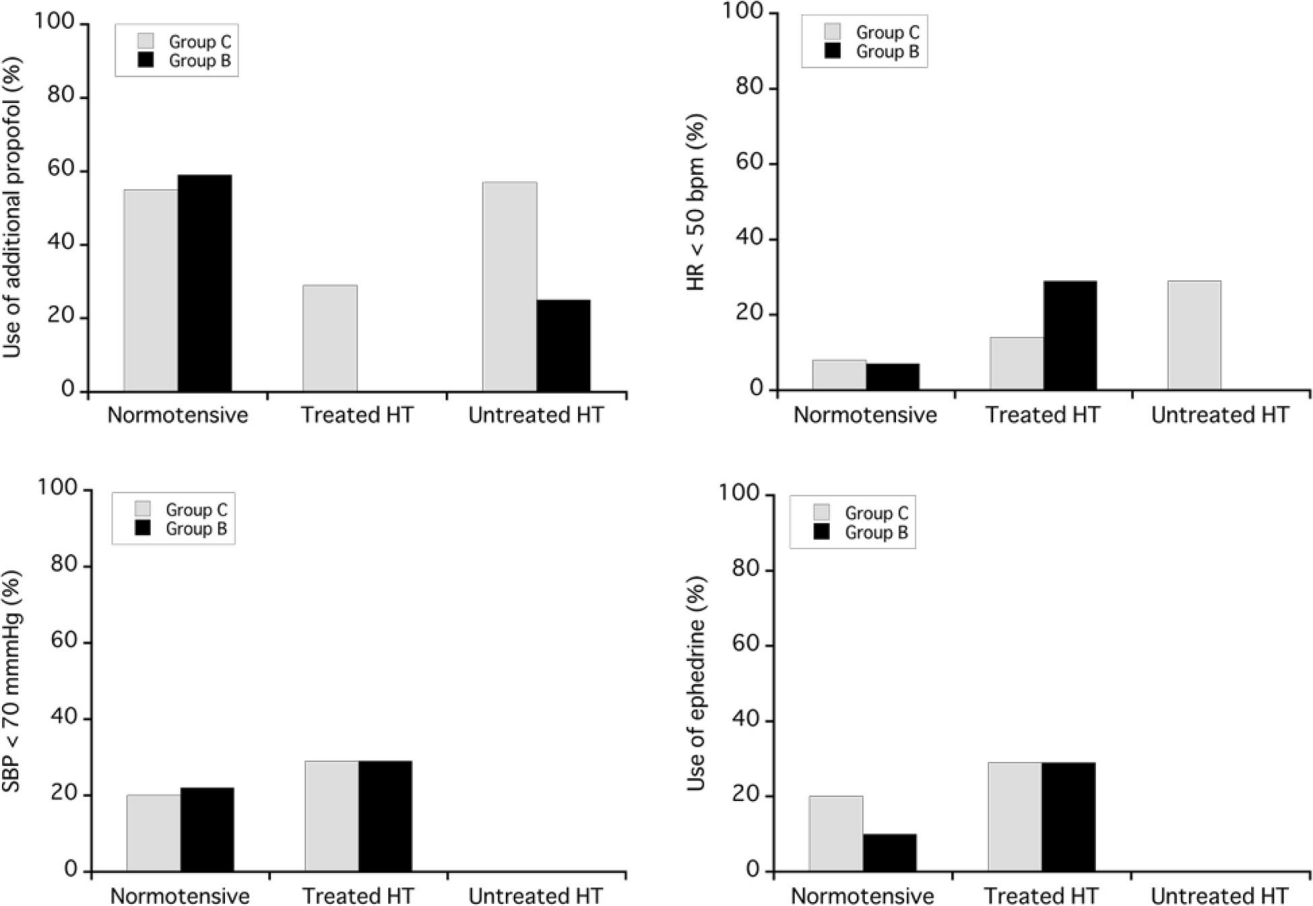
Use of additional propofol and adverse events in patients, stratified by group (%). Normotensive: patients with no history of antihypertensive medication use and with normotension, treated HT: patients with a history of antihypertensive medication use, untreated HT: patients with no history of antihypertensive medication use and with hypertension, *HT* hypertensive

Use of additional propofol and adverse events including HR < 50 bpm, SBP < 70 mmHg, and use of ephedrine are shown in Table 4 and illustrated in Fig. 5. There were no significant differences between groups in each subgroup. In normotensive patients, 55 % of patients in Group C required additional propofol, compared to 59 % of patients in Group B. Additionally, when age was categorized, patients younger than 60 years required more propofol (Group C vs. Group B: 20–29 years old: 86 % vs. 63 %, 30–39: 75 % vs. 63 %, 40–49: 56 % vs. 100 %, 50–59: 45 % vs. 55 %, over 60: 0 % vs. 14 %). In the untreated HT subgroup, SBP was never under 70 mmHg and ephedrine was never used.

**Table 4:** Use of additional propofol and adverse events in patients, stratified by group

	Group C			Group B		
Subgroups	Normotensive (*n* = 41)	Treated HT (*n* = 7)	Untreated HT (*n* = 7)	Normotensive (*n* = 41)	Treated HT (*n* = 7)	Untreated HT (*n* = 4)
Additional Propofol	22 (55 %)	2 (29 %)	4 (57 %)	24 (59 %)	0 (0 %)	1 (25 %)
Adverse Events						
HR < 50 bpm	3 (8 %)	1 (14 %)	2 (29 %)	3 (7 %)	2 (29 %)	0 (0 %)
SBP < 70 mmHg	8 (20 %)	2 (29 %)	0 (0 %)	9 (22 %)	2 (29 %)	0 (0 %)
Use of Ephedrine	8 (20 %)	2 (29 %)	0 (0 %)	4 (10 %)	2 (29 %)	0 (0 %)

The results are expressed as numbers and %. Normotensive: patients with no history of antihypertensive medication use and with normotension, treated HT: patients with a history of antihypertensive medication use, untreated HT: patients with no history of antihypertensive medication use and with hypertension, *HT* hypertensive

## Discussion

In this study, we demonstrated that a 0.3-μg/kg/min infusion of remifentanil for 5 min followed by a 0.1-μg/kg/min infusion did not differ significantly from a combination of two bolus injections of remifentanil (first bolus of 0.4 μg/kg and second bolus of 0.6 μg/kg after 3 min) and 0.1-μg/kg/min infusion of remifentanil in terms of haemodynamic responses during the induction of anaesthesia, in both normotensive patients and patients with hypertension, with or without treatment. In both remifentanil groups, HR deceased following anaesthesia induction and increased after intubation when compared to baseline HR; however, this was not significant. Blood pressure was significantly decreased from baseline after anaesthesia induction and once increased upon intubation, but again decreased from baseline after intubation. However, these changes in blood pressure were not considered clinically significant. In the treated and untreated HT patients, Group C maintained baseline blood pressure after intubation for longer than Group B.

The cardiovascular response to anaesthesia induction, laryngoscopy, and tracheal intubation may be exaggerated in the treated or untreated HT patients [8]. Maguire et al. reported that a bolus dose of remifentanil 0.5 μg/kg followed by a 0.1 μg/kg/min infusion could well control the haemodynamic response to intubation in treated HT patients. However, the increase in blood pressure was approximately 30 mmHg during this regimen, compared to a 10 mmHg increase in young adults who received a bolus dose of remifentanil 0.5 μg/kg followed by a 0.25 μg/kg/min infusion [8]. Park et al. demonstrated that a low-dose regimen of remifentanil, consisting of a 0.5 μg/kg bolus followed by a continuous infusion of 0.1 μg/kg/min, resulted in similar haemodynamic responses to induction and tracheal intubation in normotensive and treated or untreated HT patients, however untreated HT patients were shown to have a relatively large amplitude of pressure swing [9]. Our study also demonstrated that hypertensive patients had larger swings in haemodynamic response to anaesthesia induction and tracheal intubation than normotensive patients; conversely, untreated HT patients had no adverse hypotension (SBP < 70 mmHg) in either group, whereas 2 of treated HT patients (29 %) had adverse hypotension in each group. Treated HT patients might be easy to have SBP < 70 mmHg, because SBP at baseline in treated HT was lower than that in untreated HT and the haemodynamic depression in response to anaesthesia induction were not different between treated HT and untreated HT, as shown in Table 3. On the basis of these findings, the low-dose regimen of remifentanil was also shown to be effective at stabilizing haemodynamics prior to intubation, and limiting pressure responses to tracheal intubation without excessive cardiovascular depression. Besides, the remifentanil administration methods to 2 groups in this study were also low-dose regimens like those of Maguire and Park’s reports.

Remifentanil is a rapid-onset and ultra-short acting pain relief opioid. Although multiple drugs such as lidocaine [10], opioids [8, 11, 12], β-blockers [13, 14], dexmedetomidine [15] and volatile anaesthetics [16] can be used to attenuate tachycardia and hypertension due to tracheal intubation, remifentanil is considered to have the ideal pharmacological character to treat adverse haemodynamic response due to a noxious but brief stimulation of tracheal intubation. However, much higher rates of infusion or bolus doses of remifentanil than those used in our study were associated to bradycardia and/or hypotension [4, 5]. The separated bolus method of Group B may be effective in avoiding excessive cardiovascular depression before intubation.

General anaesthesia was induced with 1 mg/kg propofol and 0.6 mg/kg rocuronium, and an additional 10 mg of propofol was administrated every 10 s until there was a loss of verbal response. Although standard propofol dose at induction is 1.5–2.5 mg/kg, opioids reduce requirement dose of propofol for loss of consciousness. In combination with remifantanil, 1.0–1.5 mg propofol is sufficient for loss of consciousness [17, 18]. The need for additional propofol was similar between groups. When age was categorized, patients younger than 60 years required more propofol, suggesting that 1 mg/kg of propofol may not to be enough for anaesthesia induction in patients under 60 years old even with remifentanil administration. However, due to the individual minimum necessary amount of propofol used for the anaesthesia induction, the suppressive effect of propofol on haemodynamics may be limited in this study.

Thompson et al. reported that 50 % of patients who received 1 μg/kg of remifentanil followed by an infusion of 0.5 μg/kg/min, exhibited bradycardia (HR < 45 bpm) and hypotension (SBP < 80 mmHg), and required rescue medication [4]. In this study, adverse events such as bradycardia (HR < 50 bpm) and hypotension (SBP < 70 mmHg) were similar between groups. In both groups and subgroups, adverse events occurred in less than 30 % of patients. This lower incidence of adverse events could be related to the lower infusion regimen and the separated bolus injections.

### Limitations

The Egan model was used to estimate plasma and effect-cite concentration of remifentanil. In comparison to the Minto model [19], which is commonly used to estimate remifentanil concentration, the Egan model is not corrected for age. Hence, in the elderly, the actual remifentanil concentration can be higher than the estimated concentration. In addition, we used actual weight to determine the dose of remifentanil to be administered as it has been reported that ideal body weight-based remifentanil infusion is potentially insufficient for anaesthetic induction in obese patients [20]. The sample sizes of treated and untreated HT subgroups were small and a larger randomized study is needed to confirm these results.

## Conclusions

Both the continuous and bolus regimen of remifentanil reduced haemodynamic responses to intubation effectively, and resulted in similar haemodynamics during induction of anaesthesia within normotensive and hypertensive patients with or without anti-hypertensive medication. In other words, separated remifentanil boluses method was performed as safely as continuous infusion method and would be able to reduce time spent on anesthesia induction compared to continuous infusion.

## Abbreviations

ASA: American society of Anesthesiologists
DBP: Diastolic blood pressure
HR: Heart rate
HT: Hypertensive
MBP: Mean blood pressure
SBP: Systolic blood pressure
SD: Standard deviation

## References

[1] Egan TD, Lemmens HJ, Fiset P, Hermann DJ, Muir KT, Stanski DR, Shafer SL (1993). The pharmacokinetics of the new short-acting opioid remifentanil (GI87084B) in healthy adult male volunteers. Anesthesiology.

[2] Bürkle H, Dunbar S, Van Aken H (1996). Remifentanil: a novel, short-acting, mu-opioid. Anesth Analg.

[3] Glass PSA, Gan TJ, Howell S (1999). A review of the pharmacokinetics and pharmacodynamics of remifentanil. Anesth Analg.

[4] Thompson JP, Hall AP, Russell J, Cagney B, Rowbotham DJ (1998). Effect of remifentanil on the haemodynamic response to orotracheal intubation. Br J Anaesth.

[5] O’Hare R, McAtamney D, Mirakhur RK, Hughes D, Carabine U (1999). Bolus dose remifentanil for control of haemodynamic response to tracheal intubation during rapid sequence induction of anaesthesia. Br J Anaesth.

[6] Hall AP, Thompson JP, Leslie NA, Fox AJ, Kumar N, Rowbotham DJ (2000). Comparison of different doses of remifentanil on the cardiovascular response to laryngoscopy and tracheal intubation. Br J Anaesth.

[7] Egan TD, Minto CF, Hermann DJ, Barr J, Muir KT, Shafer SL (1996). Remifentanil versus alfentanil: comparative pharmacokinetics and pharmacodynamics in healthy adult male volunteers. Anesthesiology.

[8] Maguire AM, Kumar N, Parker JL, Rowbotham DJ, Thompson JP (2001). Comparison of effects of remifentanil and alfentanil on cardiovascular response to tracheal intubation in hypertensive patients. Br J Anaesth.

[9] Park SJ, Shim YH, Yoo JH, Nam SH, Lee JW (2012). Low-dose remifentanil to modify haemodynamic responses to tracheal intubation: comparison in normotensive and untreated/treated hypertensive Korean patients. Korean J Anesthesiol.

[10] Qi DY, Wang K, Zhang H, Du BX, Xu FY, Wang L, Zou Z, Shi XY (2013). Efficacy of intravenous lidocaine versus placebo on attenuating cardiovascular response to laryngoscopy and tracheal intubation: a systematic review of randomised controlled trials. Minerva Anestesiol.

[11] Crawford DC, Fell D, Achola KJ, Smith G (1987). Effects of alfentanil on the pressor and catecholamine responses to tracheal intubation. Br J Anaesth.

[12] Habib AS, Parker JL, Maguire AM, Rowbotham DJ, Thompson JP (2002). Effects of remifentanil and alfentanil on the cardiovascular responses to induction of anaesthesia and tracheal intubation in the elderly. Br J Anaesth.

[13] Inoue S, Tanaka Y, Kawaguchi M, Furuya H (2009). The efficacy of landiolol for suppressing the hyperdynamic response following laryngoscopy and tracheal intubation: a systematic review. Anaesth Intensive Care.

[14] Vucevic M, Purdy GM, Ellis FR (1992). Esmolol hydrochloride for management of the cardiovascular stress responses to laryngoscopy and tracheal intubation. Br J Anaesth.

[15] Kunisawa T, Nagata O, Nagashima M, Mitamura S, Ueno M, Suzuki A, Takahata O, Iwasaki H (2009). Dexmedetomidine suppresses the decrease in blood pressure during anesthetic induction and blunts the cardiovascular response to tracheal intubation. J Clin Anesth.

[16] Randell T, Seppälä T, Lindgren L (1991). Isoflurane in nitrous oxide and oxygen increases plasma concentrations of noradrenaline but attenuates the pressor response to intubation. Acta Anaesthesiol Scand.

[17] Conway DH, Hasan SK, Simson ME (2002). Target-controlled propofol requirements at induction of anaesthesia: effect of remifentanil and midazolam. Eur J Anaesthesiol.

[18] Jee YS, Hong JY (2008). Effects of remifentanil on propofol requirements for loss of consciousness in target-controlled infusion. Minerva Anestesiol.

[19] Minto CF, Schnider TW, Egan TD, Youngs E, Lemmens HJ, Gambus PL, Billard V, Hoke JF, Moore KH, Hermann DJ, Muir KT, Mandema JW, Shafer SL (1997). Influence of ageand gender on the pharmacokinetics and pharmacodynamics of remifentanil. I Model development. Anesthesiology.

[20] Kunisawa T, Mitamura S, Hanada S, Suzuki A, Takahata O, Iwasaki H (2012). Ideal body weight-based remifentanil infusion is potentially insufficient for anesthetic induction in mildly obese patients. J Anesth.

